# Effect of Adding Monohydrocalcite on the Microstructural Change in Cement Hydration

**DOI:** 10.1021/acsomega.2c03977

**Published:** 2022-10-03

**Authors:** Chaiwat Photong, Wanawan Pragot

**Affiliations:** School of Energy and Environment, University of Phayao, 19, Mae Ka, Mueang Phayao District, Phayao56000, Thailand

## Abstract

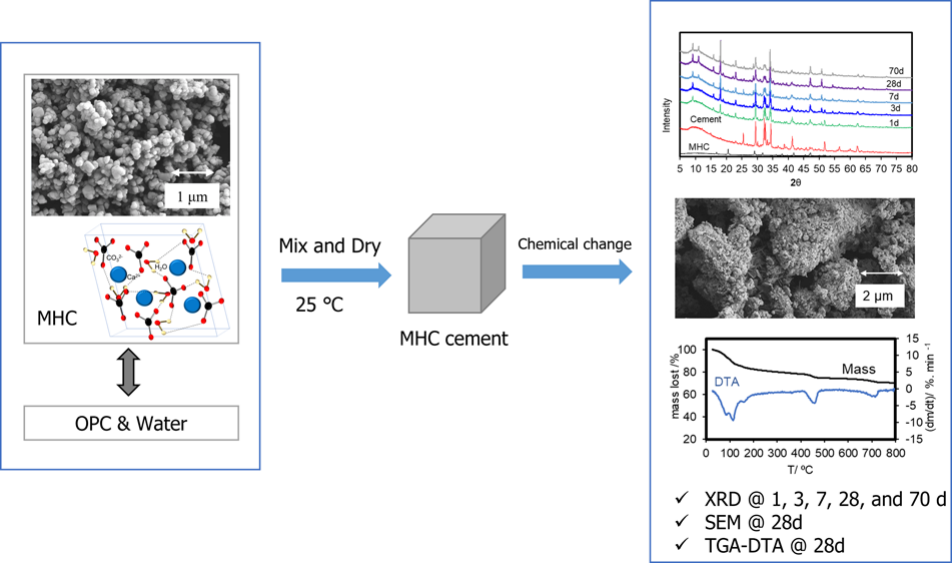

This study explored the application of mineral carbonation products in the form of monohydrocalcite (MHC) as a Portland cement additive. This work studied the effect of adding monohydrocalcite on the microstructural change in cement hydration. We investigated the hydration and microstructure development of MHC–cement at different aging times and different MHC mass % values. Chemical composition changes over time were investigated using X-ray diffraction (XRD) and scanning electron microscopy (SEM). The hydration behaviors of MHC blended with cement were monitored using thermogravimetric analysis and differential thermal analysis (TGA–DTA). The results indicated that the role of MHC in the hydrated cement was enhancing the cement hydration process with may increase the long term strength gain. We also discovered the effect of MHC in term of the long-term chemical reaction and forming the new phase formation of tilleyite in the hydrated MHC cement at long curing ages that was not present in the ordinary Portland cement (OPC) system.

## 1 Introduction

Precipitate calcium carbonate (PCC) is one of several products from the mineralization process. PCC is widely used in many different industries, and its market is estimated to reach 98.7 million metric tonnes by 2020.^1^ The global market of calcium carbonate products is estimated to grow 96 Mt/year,^1^ and the U.K. market for calcium carbonate products is 2 Mt/year.^1^ Financial incentives are not the only driving force for PCC production. With at least 37% of CO_2_ by mass, PCC offers great potential for CO_2_ sequestration. Among its numerous uses, PCC can be used as a clinker substitute for Portland cement. One attractive choice is its use in construction materials, a competitive market with a high demand. PCC may be used along with cement to produce concrete, where PCC can potentially improve certain properties such as the strength of the material rather than acting just as a filler.^2−4^ In this regard, products such as PCC produced through the mineral carbonation processes could be a promising alternative to minimize the environmental impact of the cement industry. These products could enter well-established markets with large demand such as cladding in the construction sector, thus partially replacing carbon-intensive products with mineral carbonation products.

**Figure 1:**
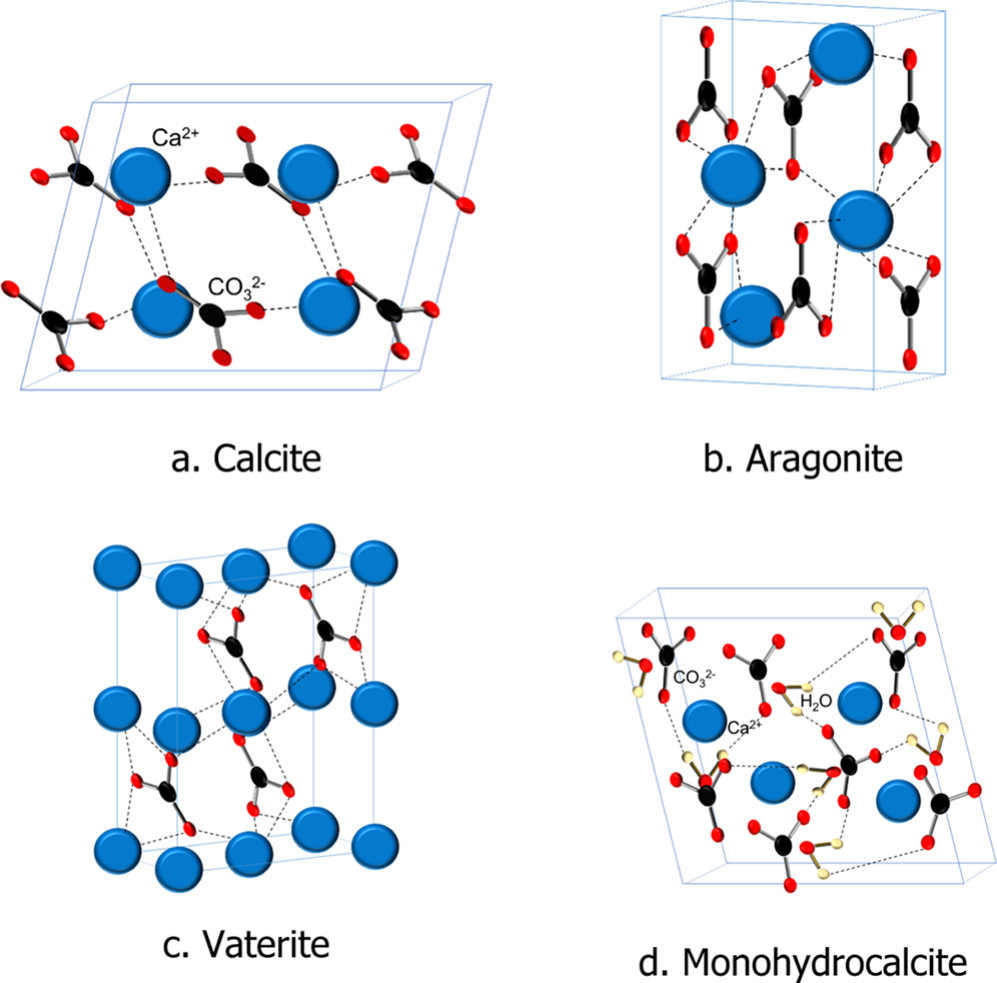
Crystal structure of CaCO_3_.

The advantage of synthetic CaCO_3_ or precipitated calcium carbonate (PCC) over CaCO_3_ produced from natural materials (ground calcium carbonate, GCC) is that PCC can be synthesized with specific requirements in mind based on several properties such as polymorphic composition, morphology, crystal size distribution, surface area, degree of whiteness, brightness, and so on.^5^ PCC generally occurs in five stable and crystalline forms, the anhydrous polymorphs (calcite, aragonite, and vaterite) and the metastable hydrated phases (monohydrocalcite, CaCO_3_·H_2_O, and ikaite, CaCO_3_·6H_2_O).^6−8^ There are three dominant dehydrated structures of CaCO_3_ named calcite, aragonite, and vaterite. Their physical properties are described in Table 1. The most usual and stable form is hexagonal calcite. Orthorhombic aragonite is less abundant, and hexagonal vaterite is the least common of the three.^9^ Calcite is precipitated at low temperature and under supersaturation conditions.^9−12^ The experimental results from Walker et al.^12^ showed aragonite formation under high pressure with an orthorhombic structure. The aragonite structure is transformed from the calcite structure. Figure 1a–c shows the structures of calcite, aragonite, and vaterite. The carbonate group of the calcite structure lies only in a single structure. In the aragonite structure, the carbonate group lies in two structural planes with the carbonate groups facing opposite directions. Moreover, it was found that temperature affects the form of calcium carbonates, with the tendency to produce aragonite under warm conditions and calcite under cold conditions. From this transformation, it can be observed that aragonite has higher specific gravity than that of calcite and it is less stable than calcite under atmospheric conditions.

**Table 1:** Calcium Carbonate Form^a^^b^^c^^d^

				physical properties		
composition	common name	stability	p*K*_sp_^d^	structure	hardness	SP
CaCO_3_	calcite	stable at atm	8.48	hexagonal^d^	3	2.71
CaCO_3_	aragonite	stable at high temperature	8.34	orthorhombic, pseudohexagonal^d^	3.5–4	2.95
CaCO_3_	vaterite	metastable	7.93	hexagonal^d^	3–4	2.95
CaCO_3_	amorphous calcium carbonate	metastable	N/A	round structure in the metastable phase^d^	none	none
CaCO_3_·H_2_O	monohydrocalcite	stable at atm	7.33	hexagonal^d^	none	2.38
CaCO_3_·6H_2_O	ikaite	stable at low temperature	6.62	monoclinic^d^	none	1.83

a atm = atmospheric temperature and pressure. SP = specific gravity.

b Solubility product constant at 20 °C in p*K*_sp_ unit (p*K*_sp_ = −log_10_).^13,14^

c Mineralogy.

d Amorphous calcium carbonate.^15,16^

Monohydrocalcite (MHC) and ikaite, CaCO_3_·6H_2_O, are hydrated CaCO_3_ polymorphs that contain water in the structural molecule. MHC can be found in environments where small concentrations of magnesium ions (Mg^2+^) in the aqueous solution of calcium ions (Ca^2+^) and carbonate ions (CO^3–^) are present, such sea water. Ikaite is not found in the nature because it is a metastable form that occurs in cold environments and easily transforms to the stable dehydrated CaCO_3_ forms such calcite, aragonite, and vaterite.

A rarely considered alternative to the most common mineralization products, such as CaCO_3_ and MgCO_3_, is the hydrated CaCO_3_ polymorph monohydrocalcite (MHC). As a mineralization product, it is interesting for several reasons. First of all, just as calcite, vaterite, and aragonite, it sequesters CO_2_ at the same CO_2_ to metal oxide ratio of 1:1. Another reason for considering MHC a mineralization product is its frequent presence in laboratory syntheses under a wide range of conditions. MHC is especially common in precipitating environments that contain calcite and inhibiting ions, such as Mg^2+^.^17−21^ It is reasonable to assume that all brines, if they are similar to (or contain) seawater, will have dissolved Mg^2+^. The presence of small concentrations of Mg^2+^ in the aqueous solution of Ca^2+^ and CO_3_^–^ and moderate pH values can enhance the transformation of CaCO_3_ into crystalline carbonate polymorphs. Therefore, Mg^2+^ plays a key role in MHC crystallization. The presence of Mg^2+^ can affect the formation of the MHC crystal lattice structure and the water on its surface such as crystal water, absorbed water, and occluded water. Small quantities of Mg^2+^ are incorporated on the MHC lattice structure and facilitate the stabilization of crystal water.^22^ The presence of water in MHC makes its structure more open and less dense compared to that of the anhydrous forms of CaCO_3_ including calcite, vaterite, or aragonite. Thus, the existence of extra water and occluded water could facilitate the increase in the strength of building blocks of MHC and have useful applications in construction.

MHC is stable at room temperature and atmospheric pressure. It has the chemical formulae CaCO_3_·H_2_O and has a hexagonal crystal structure of *P*3_1_, with *a* = *b* = 10.555 and *c* = 7.564.^23^ The crystal structure of MHC is different from that of other precipitated calcium carbonates, calcite,^6^ vaterite,^7^ and aragonite;^8^ the MHC crystal structure has structural water molecules^24^ (Figure 1). The atomic structure of MHC consists of irregular 8-folded Ca–O polyhedra, with the central Ca^2+^ surrounded by carbonate groups and structural water molecules. Therefore, there are two types of bonds inside the MHC structure: (1) these structural water and carbonate groups are bonded with ionic bonds between H and O atoms and (2) the bond between the Ca^2+^ atom and carbonate groups. However, there is only one type of bond between the Ca^2+^ atom and carbonate groups for other precipitated calcium carbonates, calcite, vaterite, and aragonite (Figure 1). Based on its solubility product (*K*_sp_) at room temperature, MHC (log *K*_sp_ = – 7.39) is more soluble in water than thermodynamically stable calcite (log *K*_sp_ = – 8.42) and metastable aragonite (log *K*_sp_ = – 8.22).

At present, MHC does not have any reported commercial applications. However, MHC has shown promising results in several studies. For instance, MHC was used in remediation to remove arsenic. This material displayed a higher adsorption capacity for arsenic than that of calcite.^20^ Furthermore, MHC shares many similarities in chemical composition, precipitation conditions, and thermal behavior to hydrated amorphous calcium carbonate (ACC),^25^ a mineral which is already widely utilized in many industries, such as the preparation of structural composite materials.^26^

There are two main mechanisms involved in the hydration of Portland cement: (1) calcium silicate hydrate (C–S–H) system and (2) calcium aluminate and sulfoaluminate system. On the one hand, in the calcium silicate hydrate system, when Portland cement is mixed with water, the cement particles hydrate and release calcium ions. Calcium and hydroxide ions in the solution react and precipitate calcium hydroxide or portlandite (Ca(OH)_2_, abbreviated as CH). At the early stage of the hydration process, amorphous silicas such as that from tricalcium silicate or alite (Ca_3_SiO_5_, abbreviated as C_3_S) and dicalcium silicate or belite (Ca_2_SiO_4_, abbreviated as C_2_S) are rapidly hydrated within a few minutes and the hydration continues for days where the equally rapid generation of C–S–H is associated with the increase of cement strength over time.^27^ C_3_S is responsible for the early strength development. 70% of C_3_S will be reacted in 28 days. C_2_S is responsible for the late strength development. Only 30% of C_2_S will be reacted in 28 days. They constitute 20–40% of cement. Therefore, C_3_S and C_2_S are responsible for the strength of cement. When C_3_S and C_2_S are mixed with water, they produce calcium hydroxide (Ca(OH)_2_, CH) and calcium silicate hydrate ((CaO)_3_(SiO_2_)_2_·4H_2_O, C–S–H).

The reactions of C_3_S and C_2_S with water (H_2_O) are provided below
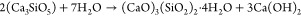
reaction 1
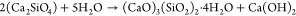
reaction 2On the other hand, in the calcium aluminate and sulfoaluminate system, ettringite (AFt, Ca_6_Al_2_(SO_4_)_3_(OH)12.26H_2_O) is the main product. Both ettringite and C–S–H can react with CO_2_ present in the atmosphere, which can lead to the formation of CaCO_3_ within the cement matrix.^28−32^ Therefore, to prevent the carbonates from being exposed to atmospheric CO_2_, the cement samples used in this study were immersed in water during the curing period.

Applications of CaCO_3_ polymorphs, primarily calcite, have been reported in numerous studies. GCC with large grain size primarily acts as an inert filler in cement due to its nonpozzolanic nature.^33^ GCC and PCC with small size can act as an inert filler and/or reactive material.^2−4,34−36^ When the amount of calcium is undersaturated, the reaction between calcium-containing phases and tricalcium aluminate (C_3_A) of the cement leads to the appearance of a family of phases known as alumina–ferrite–monosulfate (AFm) phases. When calcite is present, calcium hemicarboaluminate Ca_4_Al_2_(CO_3_)0.5(OH)_13_·5.5H_2_O (abbreviated as CaCO_3_-AFm) forms through a solid-state reaction between C_3_A and CaCO_3_. Calcite behaves as a filler when it is oversaturated and in excess. The amount of excess calcite is usually less than the total amount added because some of it will react.

Recent studies provide important insights into the structure of MHC. It has a more open and less dense structure compared to that of the anhydrous forms of CaCO_3_ such as calcite, vaterite, or aragonite because of the incorporation of structural water. This water could facilitate an increase in the strength of cement building blocks and decrease the overall water required for hydration of the cement. However, as mentioned earlier, MHC does not have any reported commercial applications and does not have any comprehensive analyses reported on its application in construction materials. Therefore, this study aimed to investigate the effect of adding MHC (monohydrocalcite) on the microstructural change in cement hydration. The study was carried out on samples made from Portland cement paste with and without MHC using water-to-solid ratios (W/C) of 0.5 to observe the hydration process of the MHC-blended cement. The formation of the chemical phases after 1, 3, 7, 28, and 70 days of curing was investigated using X-ray diffraction (XRD) to determine the phase development quantitatively (i.e., unhydrated cement, AFt, CH, C–S–H, and AFm phases). Furthermore, the study included an analysis of the microstructure of the samples. Fragments after 28 days were collected and further characterized with thermogravimetric analysis and differential thermal analysis (TGA–DTA) and scanning electron microscopy (SEM) analysis. Overall, the main objective was to study the influence of MHC on the Portland cement hydration process and therefore determine how feasible this polymorph is as a construction material.

## 2 Materials and Methods

### 2.1 Materials

#### 2.1.1 MHC Powder

**Figure 2:**
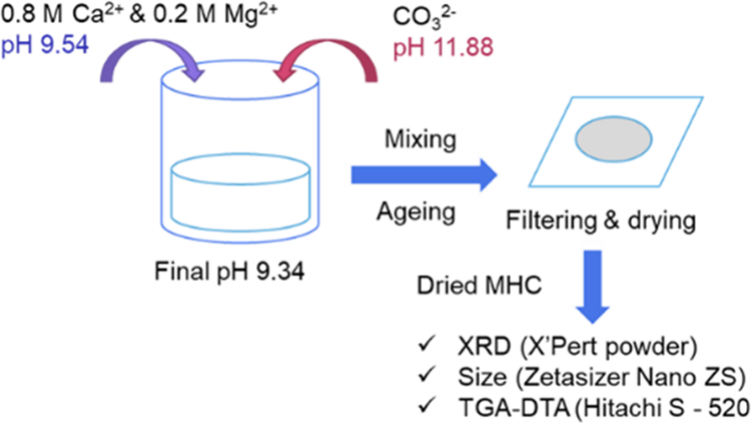
MHC preparation method.

**Figure 3:**
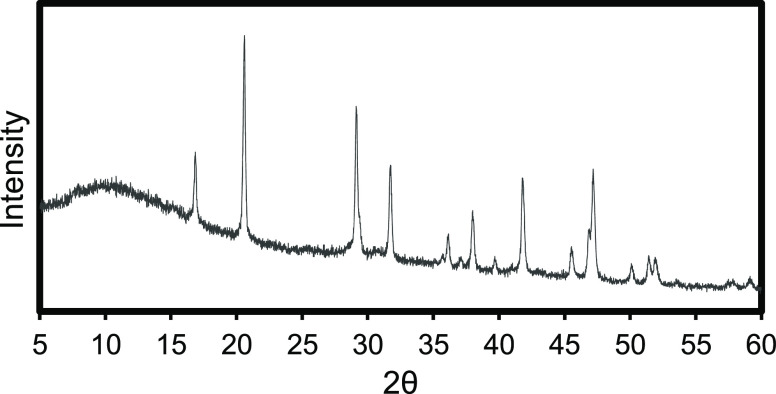
XRD pattern of pure MHC.

**Figure 4:**
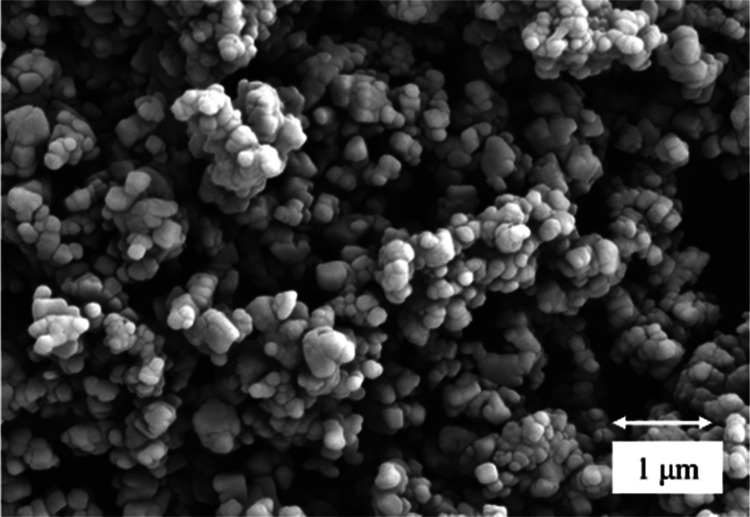
SEM of the synthesized MHC used in this experiment with nanosize varying from 80 to 500 nm.

MHC powder was synthesized in the laboratory using brine and carbonate solutions. The MHC preparation method is displayed in Figure 2. This synthesis method was decided such that synthetic brines had the same chemical constituents as those of concentrated seawater. The Mg^2+^ concentration was set at 0.2 M, taking into consideration that most of the natural brines were expected to contain at least a small amount of Mg^2+^.^21,37^^21,37^ Single-phase MHC was prepared by adding simultaneously 1 M Na_2_CO_3_ (pH 11.88) and 1 M brine containing 0.2 M MgCl_2_ and 0.8 M CaCl_2_ aqueous solution (pH 9.54). Mg^2+^ helps the crystal formation of the hydrated form MHC because the presence of Mg^2+^ can affect the formation of MHC crystal water, absorbed water, and occluded water. Small quantities of Mg^2+^ are incorporated on the MHC lattice structure and facilitate the stabilization of crystal water.^22^ After an aging time of 1 h with constant stirring, the precipitate (pH 9.34) was settled, filtered, and rinsed to remove excess NaCl salt and remove all Mg^2+^ in the solution. The filtrate was dried at 40 °C overnight, and its chemical composition was confirmed by XRD (Panalytical X’Pert Pro) that the obtained product was 100% MHC. The XRD patterns of the MHC samples were obtained using a Panalytical X’Pert Pro diffractometer with a reduced range of 5.0065–59.9962° 2θ and a scan time of 16.575 s (Figure 3). The XRD of this pure MHC is depicted in Figure 3. Crystalline MHC constituted spherical particles with crystal agglomeration as shown in Figure 4, as characterized by SEM analysis (Hitachi S-520). The size of MHC is on the nanoscale ranging from 80 to 5000 nm with an average diameter of 380.8 nm, as measured using a Zetasizer Nano ZS (Figure 4). This size of the MHC particles is smaller than the grain size of cement, which can vary from 45 to 300 μm (ASTM C430-9C430-96, ASTM C786-96).

**Figure 5:**
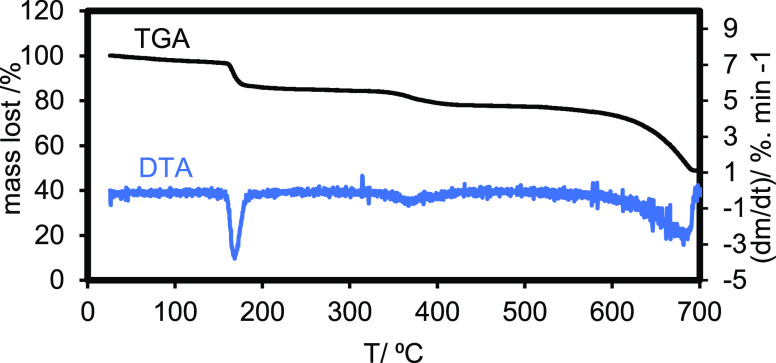
TGA–DTA of pure MHC.

The MHC thermal decomposition behavior was analyzed using thermogravimetric analysis TGA–DTA (STANTON REDCROFT, U.K.). The details of the method for characterizing the thermal decomposition behavior is provided in Section 2.4. The TGA showed a well-established two-step decomposition with a mass loss value of 9.51% at 170 °C and 9.2% at 380 °C. This was in good agreement with the appearance of the DTA endothermic peaks, indicating the MHC structure degradation (Figure 5). These values were nearly equivalent to those calculated from the following reactions: (1) CaCO_3_·H_2_O → CaCO_3_0.5H_2_O + 0.5H_2_O and (2) CaCO_3_0.5H_2_O → CaCO_3_ + 0.5H_2_O.

#### 2.1.2 Cement

In the case of cement, CEM I 52.5N OPC cement supplied by Hanson Cement UK was used. CEM I 52.5N OPC had a Blaine fineness of 395 m^2^/kg. The chemical composition and the oxide contents are given in Tables 2 and 3 that are analyzed by X-ray fluorescence and Bogue calculation.

**Table 2:** Chemical Composition of CEM I 52.5N OPC Cement

phase name^a^	cement chemistry	chemical name	formula	% mass
belite	C_2_S	calcium disilicate	Ca_2_SiO_4_	15.24
alite	C_3_S	calcium trisilicate	Ca_3_SiO_5_	59.65
calcite	CC̅	calcium carbonate	CaCO_3_	4.8
ferrite	C_4_AF	tetracalcium aluminoferrite	4CaO·Al_2_O_3_·Fe_2_O_3_	8.65
gypsum	CS̅H_2_	calcium sulfate dihydrate	CaSO_4_·2H_2_O	4.65
celite	C_3_A	tricalcium aluminate	3CaO·Al_2_O_3_	7.01

a Cement chemistry’ notation: calcium or calcium oxide (CaO) = C, silicon oxide of silica (SiO_2_) = S, aluminum oxide or alumina (AlO_3_) = A, iron oxide (Fe_2_O_3_) = F, sulfate (SO_3_) = S̅, and water (H_2_O) = H.

**Table 3:** Oxide Contents of CEM I 52.5N OPC Cement

oxides	% mass
SiO_2_	20.28
Al_2_O_3_	4.71
Fe_2_O_3_	3.27
CaO	67.13
SO_3_	2.54
MgO	0.67
K_2_O	1.40

### 2.2 Hydrated Cement Preparation Method

**Figure 6:**
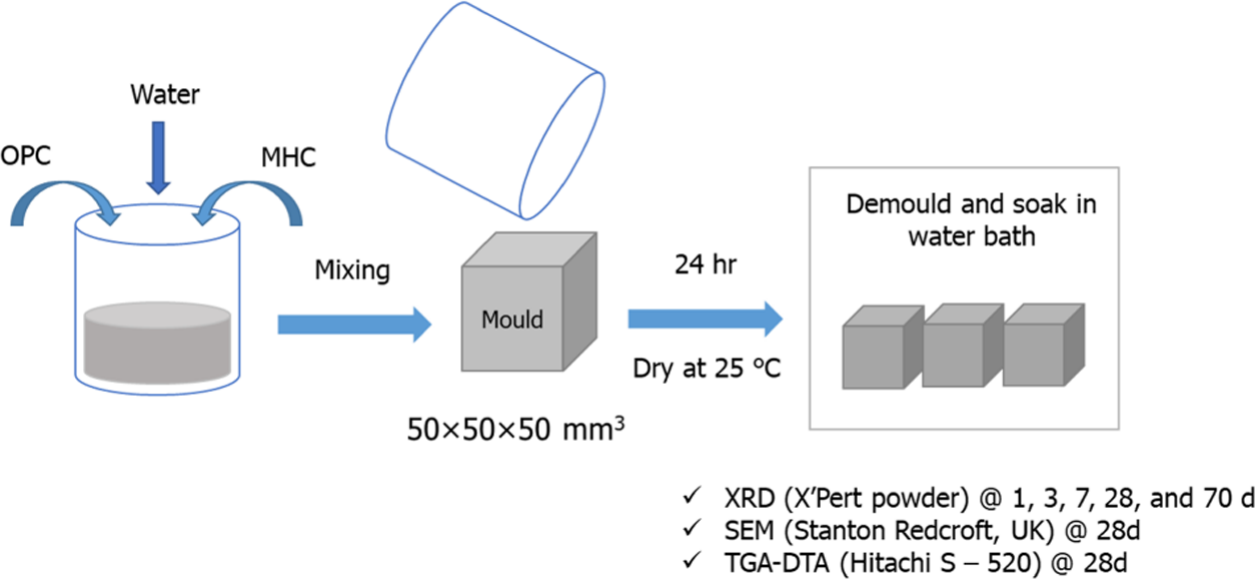
Methodology to determine the role of MHC in cement for construction application.

The cement and MHC pastes were prepared by manually mixing 600 g of total solids with the desired W/C. Initially, the cement and MHC powder were combined, and then, water was added to the solid mixture and stirred continuously for 2 min. The resulting paste was transferred to three 50 × 50 × 50 mm^3^ steel molds. The cubic molds were placed in an oven at a constant temperature of 25 °C. After a setting period of 24 h, the cement cubes were demolded and submerged in a water bath at room temperature for 1, 3, 7, 28, and 70 days to cure. The sample preparation procedure is depicted in Figure 6.

The effect of MHC concentration in hydrated cement was determined by varying the amount of MHC in the paste from 0 to 25% using a constant W/C equal to 0.5. A summary of the composition of each sample is provided in Table 4.

**Table 4:** Cement, MHC, and Water Composition Used in the Cement Hydration Study

sample	cement, g	MHC, g	water, g
control, 0% MHC cement 0.5 W/C	600	0	300
5% MHC cement 0.5 W/C	570	30	300
10% MHC cement 0.5 W/C	540	60	300
15% MHC cement 0.5 W/C	510	90	300
20% MHC cement 0.5 W/C	480	120	300
25% MHC cement 0.5 W/C	450	150	300

### 2.3 X-ray Diffraction (XRD) Analysis

The chemical composition of the hydrated cubes prepared at curing times of 1, 3, 7, 28, and 70 days was tested using XRD. The cubes were ground into fine powder before the XRD analysis. The cement and hydrated cement powder were loaded onto standard holders. The test was performed on a Panalytical X’Pert Pro diffractometer equipped with a Cu X-ray source, operating under the following conditions: 40 kV, 45 mA, temperature of 25 °C, step size of 0.0130° 2θ, range of 5.0036–80.9756° 2θ, scan time of 164 s, and continuous scan mode.

The crystalline mineral phases were identified with software X’pert HighScore Plus (PANalytical, NL) using reference patterns from the Crystallography Open Database. Additionally, quantitative analysis was done by applying the Rietveld refinement method to the recorded diffraction patterns. The peak background was removed with 5 bending factors and 10 granularities. The standard deviation is 1% based on the results from the three samples. The additional information for the phases used in the quantitative Rietveld analyses is provided in Table 5.

**Table 5:** Additional Information for the Phases Used in the Quantitative Rietveld Analyses

phase name	cement chemistry	chemical formula	ICSD^a^ code
MHC		CaCO_3_·H_2_O	100847
calcite	CC̅	CaCO_3_	80869
portlandite	CH	Ca(OH)_2_	202220
belite	C_2_S	Ca_2_SiO_4_	81097
alite	C_3_S	Ca_3_SiO_5_	94742
ferrite	C_4_AF	4CaO·Al_2_O_3_·Fe_2_O_3_	161525
calcium hemicarboaluminate (CaCO_3_-AFm)	AFm	Ca_4_Al_2_(CO_3_)0.5(OH)_13_·5.5H_2_O	41-0221
ettringite	AFt	Ca_6_Al_2_(SO_4_)_3_(OH)_12_·26H_2_O	155295
tilleyite		Ca_5_(Si_2_O_7_)(CO_3_)_2_	14256

a ICSD = Inorganic Crystal Structure Databases.

### 2.4 Thermal Behavior Characterization

Thermogravimetric analysis (TGA) and differential thermal analysis (DTA) were done using an STA 780 (Stanton Redcroft, U.K.) instrument on the hydrated cement powder cured at 28 days that was ground into a fine powder. The TGA–DTA measurement was carried out using ∼20–30 mg of the sample, weighed into a platinum crucible. The samples were subjected to a dynamic heating rate of 2 °C/min from 25 to 700 °C in a flowing N_2_ environment. An alumina sample was used as a DTA reference located in a twin-crucible disk during the characterization.

### 2.5 Scanning Electron Microscopy (SEM) Image

Prior to analysis by SEM, the samples were pretreated. The 28-day finely hydrated cement samples were dispersed onto ultrasmooth carbon tape and then sputter-coated with a gold–palladium alloy under a flowing argon atmosphere. Once the samples were ready, their morphology was captured visually under a Hitachi S-520 scanning microscope with 20 kV acceleration voltage.

## 3 Results and Discussion

### 3.1 Phase Analysis

**Figure 7:**
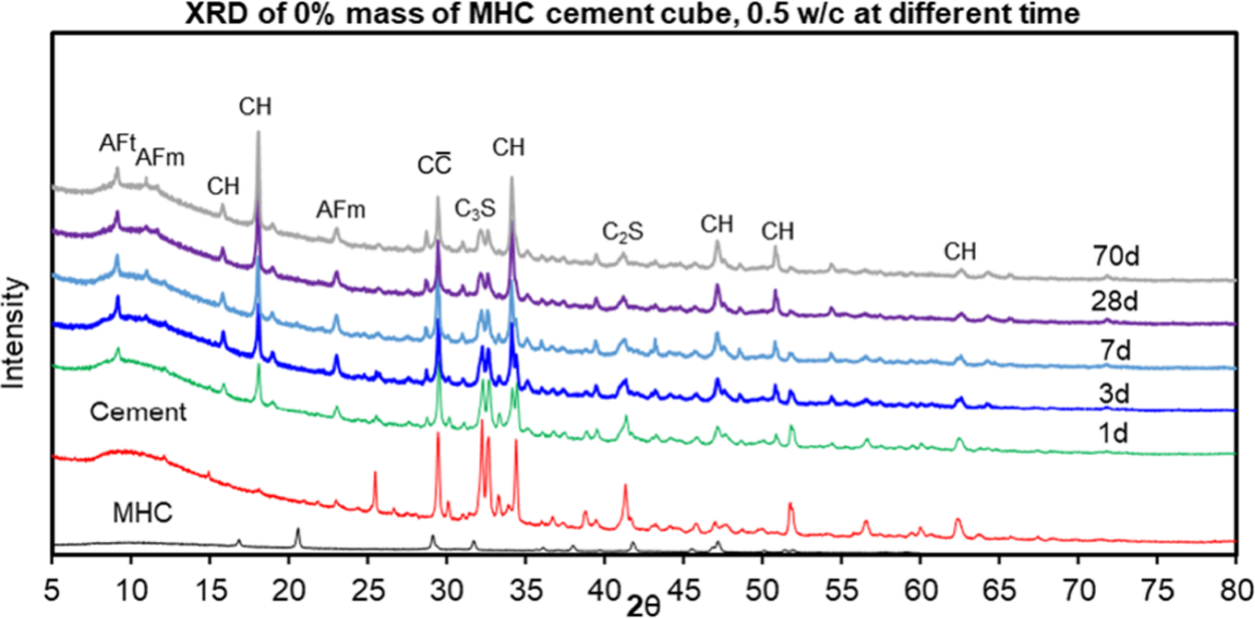
XRD pattern of hydrated control cement sample without MHC cured at 1, 3, 7, 28, and 70 days.

**Figure 8:**
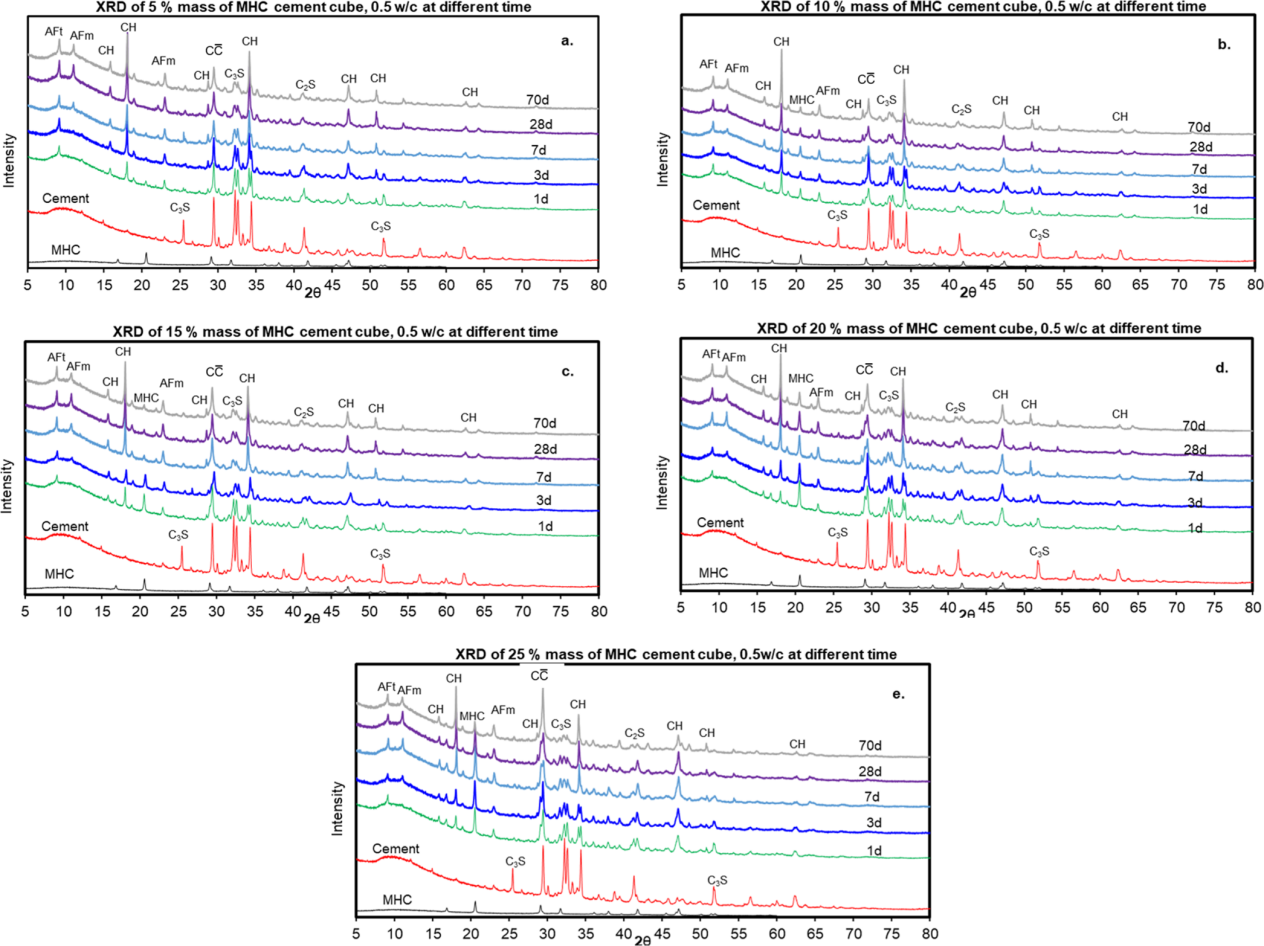
XRD pattern of 1, 3, 7, 28, and 70 days hydrate cement samples with 0.5 W/C containing MHC at (a) 5% mass of MHC, (b) 10% mass of MHC, (c) 15% mass of MHC, (d) 20% mass of MHC, (e) 25% mass of MHC.

XRD analysis was used to follow phase changes within the cement samples over time. For reference, the XRD pattern of a control sample was used. The control sample without MHC and the MHC samples cured for 1, 3, 7, 28, and 70 days are depicted in Figures 7 and 8. The MHC samples contained portlandite (CH), ettringite (AFt, Ca_6_Al_2_(SO_4_)_3_(OH)_12_·26H_2_O), belite or dicalcium silicate (C_2_S), calcium hemicarboaluminate (CaCO_3_-AFm, Ca_4_Al_2_(CO_3_)0.5(OH)_13_·5.5H_2_O), alite or tricalcium silicate (C_3_S), calcite (CC̅), and Ferrite (C_4_AF) as the main crystalline hydrate and carbonate phases as shown in Table 5.

The results of the sample without MHC defined as hydrated control cement sample without MHC after cured at 1, 3, 7, 28, and 70 days are provided in Figure 7 and Table 6. Figure 6 showed XRD pattern of hydrated control cement sample without MHC cured at 1, 3, 7, 28, and 70 days. The results of the quantitative analysis achieved with the Rietveld refinement method can be seen in Table 6. The amount displayed in the table corresponded to the percentage of each phase in the sample.

**Table 6:** Chemical Composition of the Control Samples Using 0.5 W/C of Hydrated Cement at Different Curing Time and Characterized by XRD

	curing time (days)				
phase	1	3	7	28	70
AFt	11.9	23	17.3	17.7	19.3
CH	19.2	17.3	14.1	10.7	39.1
C_2_S	18.4	16.6	14.3	15	4.8
CaCO_3_-AFm	2.9	3.5	3.7	8.7	11.1
C_3_S	37.7	22.7	17.5	20.1	0.0
CC̅	5	13.6	24.6	23.2	22.8
C_4_AF	4.9	3.3	8.5	4.7	0.0

The results indicated that after the cement mixed with water, the C_3_S and C_2_S react with water and create CH and C–S–H phase in the calcium silicate hydrate (C–S–H) system (reactions 1 and 2). These effect to the reduction of the C_3_S and C_2_S phase in the cement at longer curing time as present in Table 6. The CH decreased due to the reaction with CO_2_ in the air and carbonate in the cement to produce CC̅. When mixed with water C_3_A produce ettringite (AFt) and mono sulfate hydrate (AFm). Since C–S–H is the gel like material, the formation can be varied and give different proportion of CH, AFm, and AF during this cement hydration phase.

A reduction of the C_3_S phase paired with an increase in the intensity of the CH and AFt peaks. Due to its amorphous nature, the formation of C–S–H was not recorded using XRD analysis. The AFt phase was seen to form after 1 day of curing when the cement paste started to solidify. A maximum was reached at day 3 of curing and afterward its amount decreased steadily. This observation could be related to the reaction between the carbonate ions and ettringite^40^. We monitored the change of AFm phase in the form of calcium hemicarboaluminate (CaCO_3_-AFm). The slight increase in the intensity of the CaCO_3_-AFm peaks could be attributed to the chemical reaction between the reactive CaCO_3_ and the C_3_A of the cement. Furthermore, the relative intensity of C_4_AF hydration are similar C_3_A by forming AFt in the presence of gypsum. However, hydration this C_4_AF is much slower than hydration of C3A that confirmed by the amount remained almost the same for 28 days as shown in Table 6 and disappeared at 70 days. In cement, if there is insufficient gypsum to convert all of the C_4_AF to ettringite, then an iron-rich gel forms at the surface of the silicate particles which is proposed to slow down their hydration.

**Figure 9:**
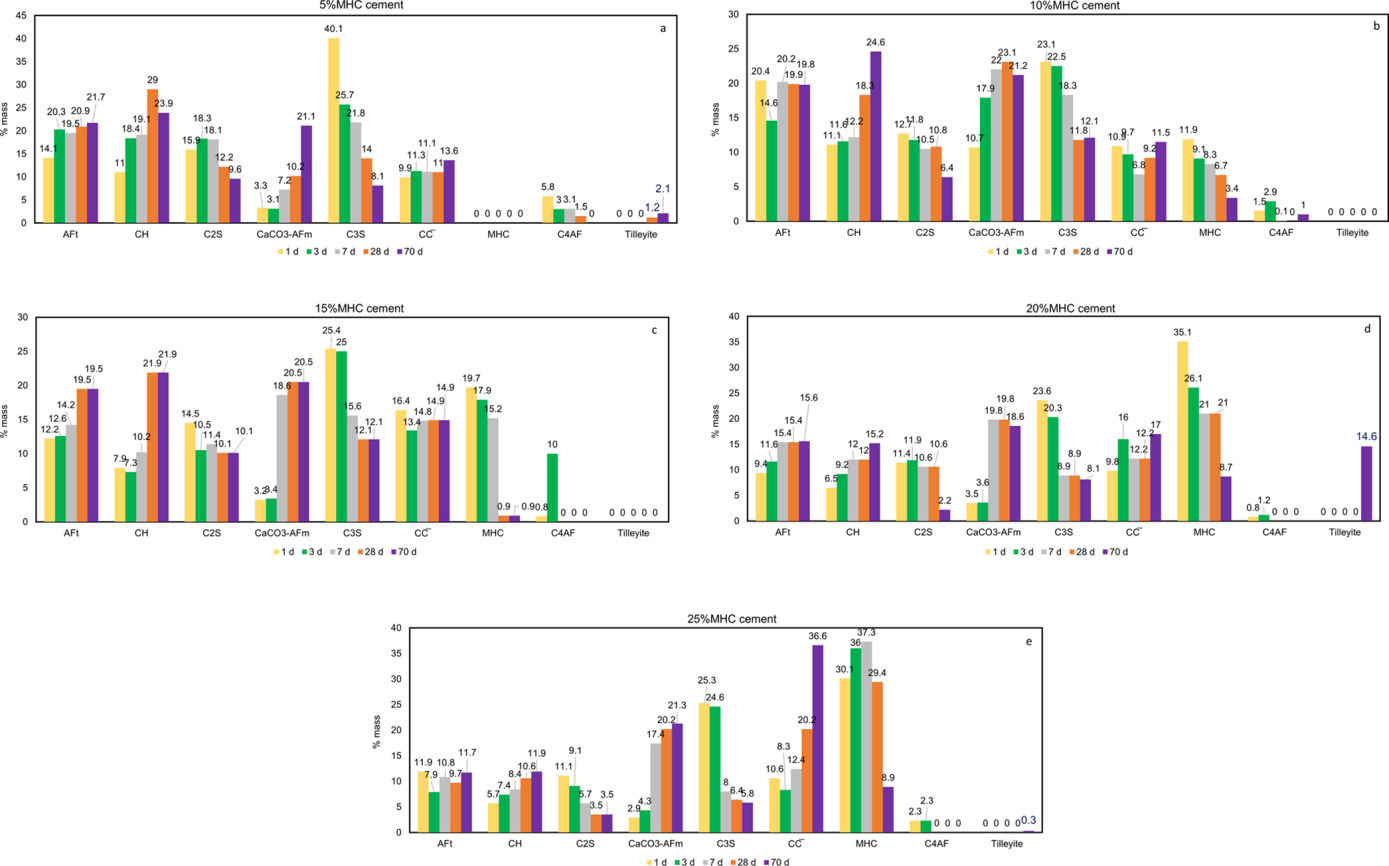
Chemical composition of the hydrated cement containing MHC prepared with 0.5 W/C at different curing times in days (d) at (a) 5% mass of MHC, (b) 10% mass of MHC, (c) 15% mass of MHC, (d) 20% mass of MHC, (e) 25% mass of MHC.

After that the effect of MHC concentration in hydrated cement was determined by varying the amount of MHC into the paste from 5 to 25% using a constant W/C equal to 0.5 as summarized the mixing in Table 4. The cement containing MHC was then analyzed the chemical composition using XRD. The XRD pattern of the hydrated cement samples containing MHC is shown in Figure 8. The samples were cured for 1, 3, 7, 28, and 70 days and contained CH, AFt, C_2_S, CaCO_3_-AFm, C_3_S, CC̅, C_4_AF, and tilleyite as part of their crystalline composition as presented. The The results of the quantitative analysis achieved with the Rietveld refinement method can be seen in Figure 9.

The hydrate cement samples with MHC has the hydration reaction. Clearly seen the reduction of the amount of C_3_S and C_2_S with time resulted to their chemical reaction with water to form other phases. This is similar to the results of the control in Table 6 and Figure 7. Another similarity with the control sampling is the amount of ferrite (C_4_AF) decreased as the curing time increased indicating the formation of C_4_AF to AFt.

Significant differences were observed between the crystalline composition of the specimens obtained from the control (Figure 7) and the hydrated cement samples containing MHC (Figures 8 and 9), in both cases at different curing times. The quantity of CH, the hydration product of cement and water, is high for hydrate cement samples containing 5 and 10% mass of MHC. For hydrate cement samples containing 15–25% mass of MHC have low quantity of CH phase. This might be possible to the calcium hydroxide hydrolyzed with the water in MHC and releases the calcium ions in released and form C–S–H gel and AFt and AFm phases. From this reason the amount of AFt and AFm phase are considerable higher for the hydrate cement samples at longer curing time as compared to the lower curring. There are two possible reasons for this. The first reason that could explain this finding is that MHC could partially react with C_3_A of the cement and transforms into CaCO_3_-AFm. Secondly, the interaction of the water structure in MHC with the C_3_A phase may have led to a more rapid formation of AFt and CaCO_3_ by donating its structural water to the cement for the cement hydration process. The MHC was consumed and the phase all disappeared for hydrate cement samples containing 5% MHC after 1 day of curing. This could be attributed to the cement hydration process and how the structural water in MHC was used and transformed into calcite. This can be observed in the amount of calcite at 1 day of curing was 9.9% mass, a value higher than the control, 5% mass. Hydrated cement samples containing initial amounts of MHC greater than 10% mass (Figure 7b–e) exhibited residual amounts and this MHC content was reduced considerably with the curing time. This was possible because the hydrated samples were oversaturated with MHC, and for this reason MHC acted as a filler. The role of MHC as a reactive chemical was detected by the increase in the quantity of the hydration and carbonation products such as AFt, CH, CaCO_3_-AFm, and CC̅ if we compare these compositions with the ones obtained from the control samples. Therefore, MHC enhanced the rate of hydration of the cement samples. Moreover, a faster rate of formation of CaCO3-AFm is evidenced due to the interaction of MHC with calcium aluminate hydrates of cement pastes and the same pattern is also found in other studies of limestone and cement.^2,3,33−35,38−41^ In that case, as the residual water of from MHC are still available, hydration continues and the induction period gives way to the third phase of hydration, the acceleratory period and this might lead to the long term strength gain or the other chemical reaction and a new phase formation.^2,40,41^ From that reason, this study found that the samples containing an initial mass of MHC has another interesting observation of the formation of tilleyite, chemical formula Ca_5_(Si_2_O_7_)(CO_3_)_2_, a new additional phase of tilleyite in some MHC cement samples (Figure 9a,d,e) at longer curing times, not observed in the control samples (sample without MHC). The slightly tilleyite peaks, at the position of 29.0803, 29.0804, 29.6923, 29.7677, 30.3202, 43.0853, and 49.1523° 2θ, were accelerated with other phases and quantity was analyzed using the software X’pert HighScore Plus (PANalytical, NL) using reference patterns from the Crystallography Open Database. The phase tilleyite was measured at 28 and 70 days of curing where it was formed out of the reaction between the cement and the residual MHC after calcite transformation at longer curing times for some hydrated cement with MHC samples, for example in hydrated cement with 5% mass of MHC samples (Figure 9a) contained tilleyite 1.2% mass for 28 days of curing and 2.1% mass for 70 days of curing. This tilleyite phase is uncommon and also reported the as the novels phase from the amorphouse calcium carbonate, nano scale calcite, and micro scale calcite chemically reaction with C–S–H in the cement during hydration reaction of cement.^40,41^ These studies indicated that the using of nano-size calcium carbonate blended cement effect to the chemical formation of tilleyite in the hydrated mineralogy.

### 3.2 Thermal Decomposition Analysis

A comparison between the chemical phases formed in the cement samples cured for 28 days and their thermal degradation was done using TGA–DTA. The thermal characterization was based on mass losses at specific temperature ranges. The temperature range between 25 and 300 °C induces the decomposition of physisorbed moisture, C–S–H, AFt and AFm. In the temperature range of 300–500 °C, CH decomposes in cement pastes containing carbonated material. Thus, the mass loss between 25 and 500 °C can be used to identify the total dehydration of the hydrated cement products. As the temperature continues to rise, the mass lost after 500 °C is attributed to the decarbonization of calcium carbonate and indicates the loss of bonded CO_2_ on the hydrated cement samples.^42^ The temperature for decarbonization can be used to differentiate the crystalline calcium carbonates. When the carbonate decomposes from 550 to 720 °C this generally suggests a poor-crystalline phase and its decomposition from 720 to 960 °C indicates a well-crystalline phase.^43^

**Figure 10:**
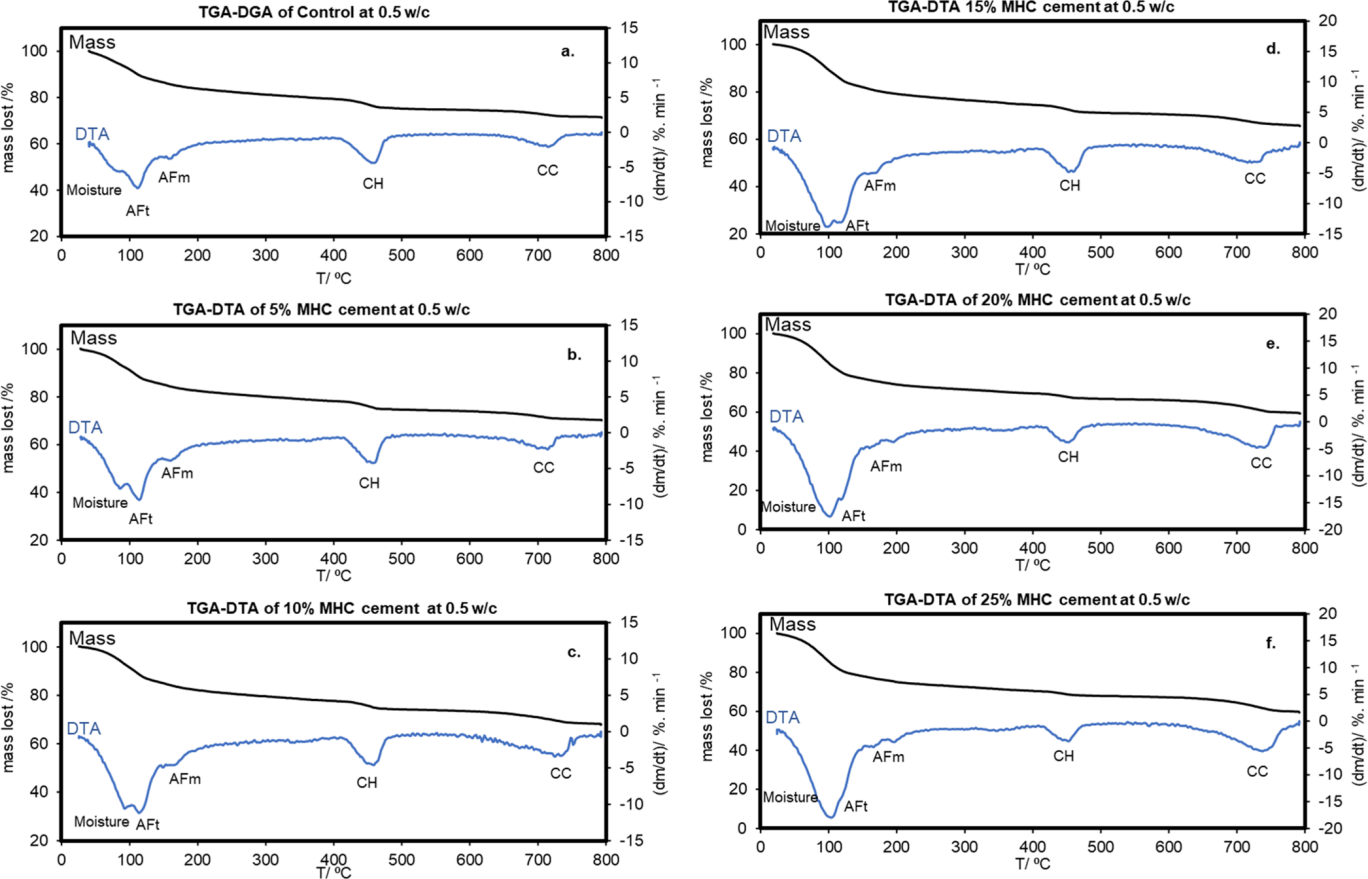
TGA–DTA of hydrated cement samples with 0.5 W/C and containing different amounts of MHC: (a) 0% mass of MHC, (b) 5% mass of MHC, (c) 10% mass of MHC, (d) 15% mass of MHC, (e) 20% mass of MHC, (f) 25% mass of MHC.

Figure 10 presents the TGA–DTA results of the hydrated MHC cement samples containing 0.5 W/C and the curing time was 28 days. The effect of the MHC concentration in the cement samples was measured using different mass percentages. The TGA curves of every sample showed a decomposition occurring in 3 steps: the first step at 100 °C, followed by a second step at 450 °C and a last step of mass lost at 750 °C. The loss of water at 25–500 °C corresponded to the moisture content whereas the mass loss after 500 °C related to the CO_2_ loss.^44^

**Figure 11:**
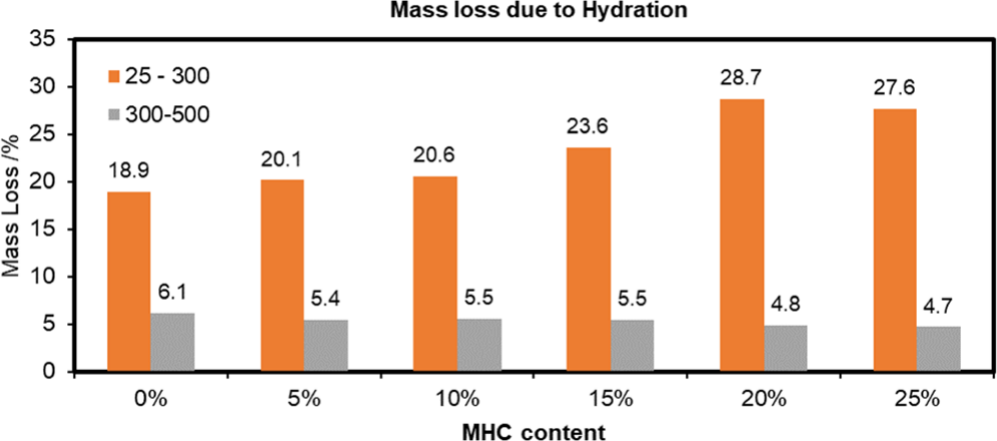
Water loss in the cement sample with/without MHC at 0.5 W/C.

**Figure 12:**
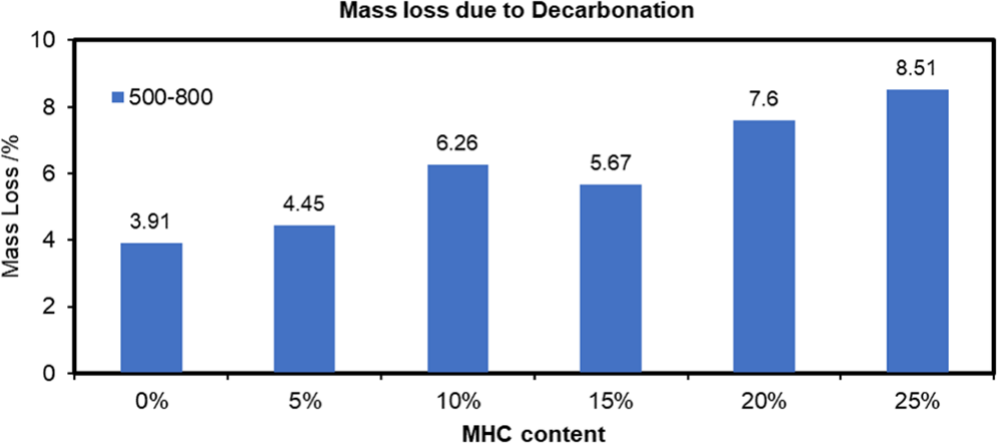
CO_2_ loss in the hydrated cement with/without MHC at 0.5 W/C.

The DTA results describe the endothermic thermal degradation of cement products with and without MHC. A comparison between control samples and samples using 5–25% mass of MHC (Figure 10) was done using DTA characterization at a broad temperature range. Results indicated a sharper reduction of DTA in the samples containing MHC cement (Figure 10b–10f), not seen in the control (Figure 10a). This points out that even a small amount of MHC in the cement is enough to affect the structure of the binding phase. At a temperature range between 25 and 200 °C, three endothermic DTA peaks were found. The three components from the thermal degradation in the region under study were:^30^ (1) structural water, (2) AFt, and (3) AFm. The DTA of structural water and AFt was marked by the prominent DTA endothermic peak at a temperature range of 25 and 150 °C. This peak was greater in the cement samples containing higher amounts of MHC indicating that MHC leads to the formation of more phases containing structural water and AFt. The amount of ettringite in the samples was greater than the measured structural water in the hydrated cement products containing 0–10% mass of MHC. However, this trend was reversed when the amount of MHC in the sample was above 15% mass (more structural water than ettringite was observed). This later case is more favorable. The excess in structural water is useful for the long-term formation of cement hydrate products because it can ensure that the water is sufficient for the hydration process.^45^ The small endothermic DTA degradation peak of AFm was observed between 150 and 200 °C. No apparent differences were observed in this AFm peak with variations in the MHC content. The second step mass loss step between 450 and 500 °C coincides with an endothermic peak is the CH degradation indicating the structural thermal degradation of hydroxy group on CH. The calcium carbonate degradation appeared during the last mass loss at 750 °C. The carbonated decomposition took place at high temperatures illustrates the possible high thermal stability and high crystallinity of the calcium carbonate phase. The decomposition of the hydration products is summarized in Figure 11. The mass of CO_2_ lost due to decarbonation is summarized in Figure 12.

Figure 11 shows the mass loss between 25 and 500 °C in the hydrated cement products under the presence and absence of MHC and with a constant water content (0.5 W/C). The mass loss between 25 and 300 °C represented the amount of structural water, AFt and AFm. Their increase was positively correlated with the addition of higher amounts of MHC in the cement preparation. On the contrary, the amount of CH decreased with an increase in the MHC content. These two trends were in good agreement with the DTA characterization.

Figure 12 depicts the effect of the initial amount of MHC in the hydrated cement samples (0.5 W/C) on the CO_2_ loss content. It is self-evident that cement samples containing more MHC showed a greater carbon content compared to the control sample where this calcium carbonate polymorph was absent. The two possible routes of calcium carbonate formation within the sample could be: (1) the transformation of C–S–H and CH into CaCO_3_, and (2) the formation of MHC when its structural water participated in the cement hydration process.

Addition of MHC influenced the hydration and carbonation reactions of hydrated cement sample and revealing the role of MHC as the reactive material. The filler effect of MHC is associated with its particle size (size of 380.8 nm). The finer MHC is related to higher packing density. The reactive effect is influenced by the amount of residual MHC in the hydrated MHC blended cement sample. TGA–DTA analysis revealed that the introduction of MHC tended to increase the formation of hydration components (especially C–S–H). As the hydration component is the main factor to the long term compressive strength gain in the cement,^2,4,28,30,36,41,46,47^ this MHC adding could be influence to the compressive strength of the hydrated cement. Substitute of cement with MHC decreases the water to react with cement particles, while the chemical effect may resulted in the increase of strength and new phase forming (tillyite).

### 3.3 SEM

**Figure 13:**
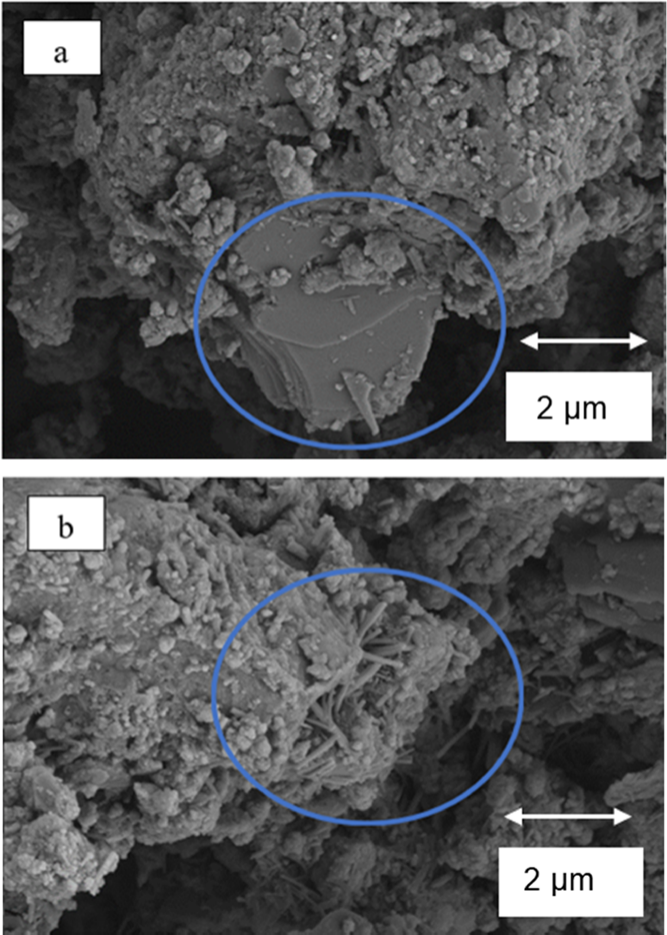
SEM of control at 28 days curing time showing the distinctive shapes of (a) portlandite (CH) and (b) ettringite (AFt).

**Figure 14:**
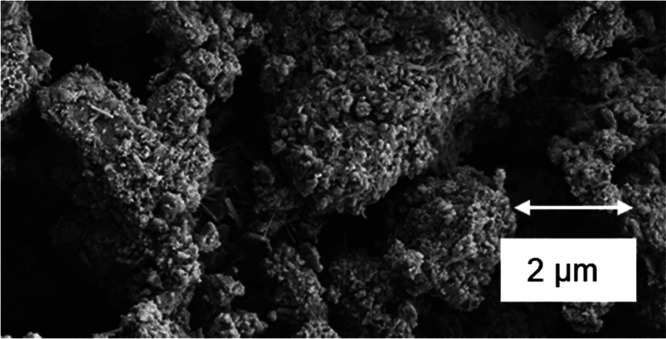
SEM showing some of ettringite and portlandite structure of a sample with 20% mass of MHC cured at 28 days.

**Figure 15:**
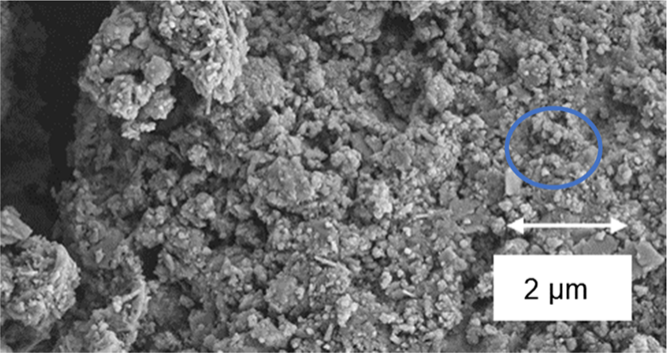
SEM showing some of ettringite, portlandite and small size calcite structure in a cement sample with 20% mass of MHC at 28 days of curing.

SEM characterization was performed on samples with different MHC contents. Results revealed four distinctive structures on the surface of the hydrated cement samples: plate-shaped, cloud like, needles, and spherical structures.^48^ Plate-shaped structures were identified as calcium hydroxide or portlandite (Figure 13a). Cloud-like structures were assigned to the C–S–H phase. Needle-like and spherical structures were found to be ettringite (Figure 13b). After 28 days of hydration, C–S–H was the dominant phase as suggested by the cloud like structure covering all the particles (Figures 14 and 15) with some calcium hydroxide was detected in each sample. The SEM analysis corroborated the formation of the hydration phases already identified by the XRD and TGA–DGA characterizations.

## 4 Conclusions

An effect of adding monohydrocalcite on the microstructural change in cement hydration was studied in this paper. The results could be applied for the use of MHC on a commercial scale as MHC proved to be an alternative product for cement clinker substitution as it showed a dual role in the hydrated cement paste, both as filler and as a reactive material. The MHC acts as filler when the quantity added is more than 10% of the mass of the cement sample resulting in the oversaturation of MHC in the sample after curing. The addition of MHC enhanced the rate of hydration reactions of cement constituents and lead to the formation of tilleyite, a rarely reported hydration product at ambient temperatures. Replacement of cement with MHC increased the water available to react with cement particles that could be beneficial for the long-term strength development and reduce the water required for complete hydration. The MHC could have potential benefits on its applicability in the construction sector and seems advantageous based on the current findings about of the effect of adding monohydrocalcite (MHC) on the microstructural change in cement hydration the chemical composition changes of cement. As the compressive strength and flexural strength are the importance parameters for the cement mechanical properties, the future work should be undertaken on the effect of addition of calcite monohydrate.
