# Correction to “Effect of Adding Monohydrocalcite on the Microstructural Change in Cement Hydration”

**DOI:** 10.1021/acsomega.2c08229

**Published:** 2023-02-08

**Authors:** Wanawan Pragot, Ara Carballo-Meilan, Lewis McDonald, Chaiwat Photong, Waheed Afzal

**Affiliations:** † School of Engineering, University of Aberdeen, King’s College, Aberdeen AB24 3UE, United Kingdom; ‡ School of Energy and Environment, University of Phayao, 19, Mae Ka, Mueang Phayao District, Phayao Thailand, 56000

The authorship has changed: Wanawan Pragot regrettably published the work that she carried out during her Ph.D. at the University of Aberdeen under the supervision of Waheed Afzal where this work had been assisted by (then) post-doc Ara Carballo-Meilan and (then Ph.D. student) Lewis McDonald. Wanawan Pragot regrets her action and wants to correct it. She notes that Chaiwat Photong helped her in proofreading the manuscript before she submitted. We believe that the contribution of Chaiwat Photong does not merit being the first author. The revised order and new additions reflect the contributions.

The Acknowledgment and Author contributions have also been revised as given here.

